# Ethyl­enediammonium bis­(3,4-dihy­droxy­benzoate) monohydrate

**DOI:** 10.1107/S1600536810042182

**Published:** 2010-10-23

**Authors:** Li-Cai Zhu

**Affiliations:** a School of Chemistry and Environment, South China Normal University, Guangzhou 510631, People’s Republic of China

## Abstract

In the title compound, C_2_H_10_N_2_^2+^·2C_7_H_5_O_4_^−^·H_2_O, the cation lies on a centre of symmetry. The crystal structure is stabilized by various inter­molecular O—H⋯O and N—H⋯O hydrogen bonds, and by weak π–π stacking inter­actions with centroid–centroid distances between symmetry-related benzene rings ranging from 3.5249 (13) to 3.7566 (14) Å.

## Related literature

For protocatechuic acid (3,4-dihydroxybenzoic acid) and its pharmacological activity, see: An *et al.* (2006 ▶); Guan *et al.* (2006 ▶); Lin *et al.* (2009 ▶); Tseng *et al.* (1998 ▶); Yip *et al.* (2006 ▶). For related structures, see: Mazurek *et al.* (2007 ▶); Xu *et al.* (2008 ▶).
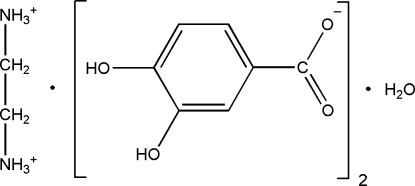


## Experimental

### 

#### Crystal data

C_2_H_10_N_2_^2+^·2C_7_H_5_O_4_^−^·H_2_O
*M* *_r_* = 386.36
Triclinic, 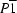

*a* = 6.8489 (8) Å
*b* = 10.7999 (12) Å
*c* = 12.0137 (13) Å
α = 75.866 (1)°
β = 81.387 (2)°
γ = 83.599 (1)°
*V* = 849.40 (16) Å^3^
*Z* = 2
Mo *K*α radiation
μ = 0.13 mm^−1^
*T* = 296 K
0.30 × 0.28 × 0.25 mm

#### Data collection

Bruker APEXII area-detector diffractometer
Absorption correction: multi-scan (*SADABS*; Sheldrick, 1996 ▶) *T*_min_ = 0.963, *T*_max_ = 0.969
4432 measured reflections
3011 independent reflections
2215 reflections with *I* > 2σ(*I*)
*R*_int_ = 0.018

#### Refinement

*R*[*F* ^2^ > 2σ(*F* ^2^)] = 0.044
*wR*(*F* ^2^) = 0.112
*S* = 1.02
3011 reflections
256 parameters
3 restraints
H atoms treated by a mixture of independent and constrained refinement
Δρ_max_ = 0.38 e Å^−3^
Δρ_min_ = −0.22 e Å^−3^

### 

Data collection: *APEX2* (Bruker, 2007 ▶); cell refinement: *SAINT* (Bruker, 2007 ▶); data reduction: *SAINT*; program(s) used to solve structure: *SHELXS97* (Sheldrick, 2008 ▶); program(s) used to refine structure: *SHELXL97* (Sheldrick, 2008 ▶); molecular graphics: *XP* in *SHELXTL* (Sheldrick, 2008 ▶); software used to prepare material for publication: *SHELXL97*.

## Supplementary Material

Crystal structure: contains datablocks I, global. DOI: 10.1107/S1600536810042182/su2221sup1.cif

Structure factors: contains datablocks I. DOI: 10.1107/S1600536810042182/su2221Isup2.hkl

Additional supplementary materials:  crystallographic information; 3D view; checkCIF report

## Figures and Tables

**Table 1:** Hydrogen-bond geometry (Å, °)

*D*—H⋯*A*	*D*—H	H⋯*A*	*D*⋯*A*	*D*—H⋯*A*
N1—H1*B*⋯O2^i^	0.89	2.04	2.904 (3)	163
N1—H1*C*⋯O4^ii^	0.89	1.94	2.803 (3)	163
N1—H1*D*⋯O4	0.89	1.98	2.741 (3)	143
N2—H2*B*⋯O3^iii^	0.89	2.09	2.931 (3)	158
N2—H2*B*⋯O6^iii^	0.89	2.54	3.079 (3)	120
N2—H2*C*⋯O8	0.89	1.90	2.742 (2)	157
N2—H2*D*⋯O7^iv^	0.89	1.94	2.799 (3)	163
O1—H1*A*⋯O1*W*^v^	0.82	1.94	2.753 (3)	169
O2—H2*A*⋯O7^vi^	0.82	1.95	2.755 (2)	168
O5—H5*A*⋯O3	0.82	2.07	2.834 (2)	156
O5—H5*A*⋯O4	0.82	2.35	3.014 (3)	139
O6—H6⋯O1*W*^vii^	0.82	1.90	2.686 (2)	160
O1*W*—H1*W*⋯O3	0.85 (2)	1.86 (2)	2.676 (2)	159 (3)
O1*W*—H2*W*⋯O8^vi^	0.88 (2)	1.85 (2)	2.725 (2)	173 (3)

Symmetry codes: (i) 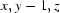; (ii) 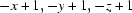; (iii) 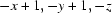; (iv) 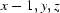; (v) 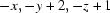; (vi) 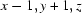; (vii) 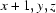.

**Fig. 1.**
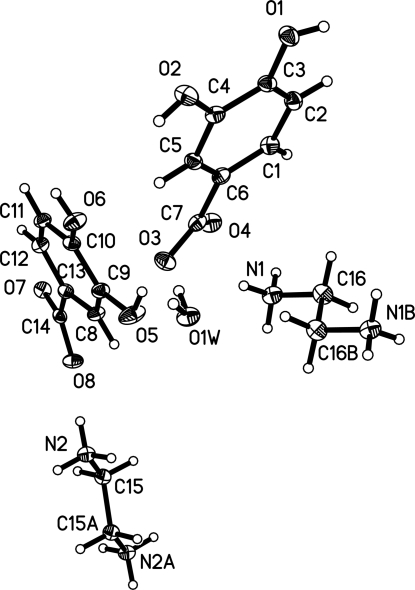
The molecular structure of the title compound showing the atomic-numbering scheme and displacement ellipsoids drawn at the 30% probability level. Symmetry codes: (A) 1 - x, -y, -z; (B) -x, 1 - y, 1 - z.

**Fig. 2.**
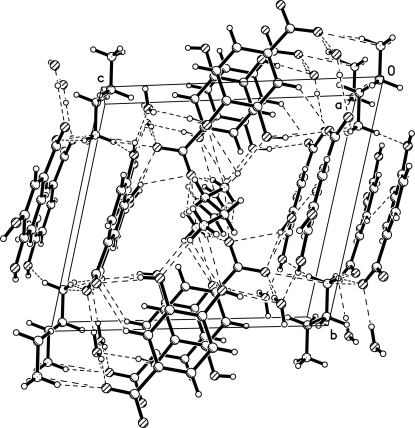
The crystal packing of the title compound showing the intermolecular hydrogen bonding interactions as broken lines (see Table 1 for details).

## supplementary crystallographic information

### Comment

Protocatechuic acid (3,4-dihydroxybenzoic acid) is one of the main secondary metabolites in the plant kingdom (Guan *et al.*, 2006). Significantly, it has been found that protocatechuic acid and its derivatives possess diverse pharmacological activities such as, antioxidant, antiapoptosis, anticarcinogen, anticoagulatory and antiinflammatory (An *et al.*, 2006; Lin *et al.*, 2009; Tseng *et al.*, 1998; Yip *et al.*, 2006). Herein, we report on the molecular and crystal structure of the title compound.

In the asymmetric unit of the title compound, illustrated in Fig. 1, there are two half protonated ethylenediammonium cations located about inversion centers, two singly deprotonated 3,4-dihydroxybenzoate anions, and one water molecule of crystallization. The bond distances and angles in the title compound are normal (Mazurek *et al.*, 2007; Xu *et al.*, 2008).

In the crystal the cations and anions are self-assembled by various intermolecular O—H···O and N—H···O hydrogen bonds (Table 1 and Fig. 2) to form a supramolecular network. The crystal structure is further stabilized by weak π-π stacking interactions (Fig. 2) occurring between adjacent benzene rings, with centroid-to-centroid distances of 3.5249 (13) Å [Cg1···Cg1^i^; Cg1 = centroid of ring (C1-C6); symmetry code (i) = 1-x, 2-y, 1-z], 3.7165 (13) Å [Cg1···Cg1^ii^; Cg1 = centroid of ring (C1-C6); symmetry code (ii) = -x, 2-y, 1-z] and 3.7566 (14) [Cg2···Cg2^iii^; Cg2 = centroid of ring (C8-C13); symmetry code (iii) = 2-x, 1-y, -z] .

### Experimental

A solution of ethylenediamine (1 mmol in 0.2 ml water) was added dropwise to a solution of protocatechuic acid (2 mmol) in acetonitrile (15 ml), and the mixture was stirred for 30 min at RT. After several days colourless block-like crystals, suitable for X-ray diffraction analysis, were obtained by slow evaporation of the solution.

### Refinement

The water molecule H-atoms were located in difference Fourier maps and were refined distance restraints of O—H = 0.86 Å and *U*_iso_(H) = 1.5*U*_eq_(O). All other H atoms were positioned geometrically and refined as riding: N—H = 0.89 Å, O—H = 0.82 Å, and C—H = 0.93 and 0.97 Å for CH and CH_2_ H-atoms, respectively, with *U*_iso_(H) = k × U_eq_(parent N, O or C-atom), with k = 1.2 for CH and CH_2_ H-atoms, and k = 1.5 for the NH_3_^+^ and OH H-atoms.

### Crystal data

**Table:**

C_2_H_10_N_2_^2+^·2C_7_H_5_O_4_^−^·H_2_O	*Z* = 2
*M**_r_* = 386.36	*F*(000) = 408
Triclinic, *P*1	*D*_x_ = 1.511 Mg m^−^^3^
Hall symbol: -P 1	Mo *K*α radiation, λ = 0.71073 Å
*a* = 6.8489 (8) Å	Cell parameters from 1194 reflections
*b* = 10.7999 (12) Å	θ = 2.9–24.2°
*c* = 12.0137 (13) Å	µ = 0.13 mm^−^^1^
α = 75.866 (1)°	*T* = 296 K
β = 81.387 (2)°	Block, colourless
γ = 83.599 (1)°	0.30 × 0.28 × 0.25 mm
*V* = 849.40 (16) Å^3^	

### Data collection

**Table:**

Bruker APEXII area-detector diffractometer	3011 independent reflections
Radiation source: fine-focus sealed tube	2215 reflections with *I* > 2σ(*I*)
graphite	*R*_int_ = 0.018
φ and ω scan	θ_max_ = 25.2°, θ_min_ = 1.8°
Absorption correction: multi-scan (*SADABS*; Sheldrick, 1996)	*h* = −8→8
*T*_min_ = 0.963, *T*_max_ = 0.969	*k* = −11→12
4432 measured reflections	*l* = −7→14

### Refinement

**Table:**

Refinement on *F*^2^	Primary atom site location: structure-invariant direct methods
Least-squares matrix: full	Secondary atom site location: difference Fourier map
*R*[*F*^2^ > 2σ(*F*^2^)] = 0.044	Hydrogen site location: inferred from neighbouring sites
*wR*(*F*^2^) = 0.112	H atoms treated by a mixture of independent and constrained refinement
*S* = 1.02	*w* = 1/[σ^2^(*F*_o_^2^) + (0.0467*P*)^2^ + 0.4204*P*] where *P* = (*F*_o_^2^ + 2*F*_c_^2^)/3
3011 reflections	(Δ/σ)_max_ < 0.001
256 parameters	Δρ_max_ = 0.38 e Å^−^^3^
3 restraints	Δρ_min_ = −0.22 e Å^−^^3^

### Special details

**Table:**

Geometry. All e.s.d.'s (except the e.s.d. in the dihedral angle between two l.s. planes) are estimated using the full covariance matrix. The cell e.s.d.'s are taken into account individually in the estimation of e.s.d.'s in distances, angles and torsion angles; correlations between e.s.d.'s in cell parameters are only used when they are defined by crystal symmetry. An approximate (isotropic) treatment of cell e.s.d.'s is used for estimating e.s.d.'s involving l.s. planes.
Refinement. Refinement of *F*^2^ against ALL reflections. The weighted *R*-factor *wR* and goodness of fit *S* are based on *F*^2^, conventional *R*-factors *R* are based on *F*, with *F* set to zero for negative *F*^2^. The threshold expression of *F*^2^ > σ(*F*^2^) is used only for calculating *R*-factors(gt) *etc*. and is not relevant to the choice of reflections for refinement. *R*-factors based on *F*^2^ are statistically about twice as large as those based on *F*, and *R*- factors based on ALL data will be even larger.

### Fractional atomic coordinates and isotropic or equivalent isotropic displacement parameters (Å^2^)

**Table:**

	*x*	*y*	*z*	*U*_iso_*/*U*_eq_	
C1	0.2895 (3)	0.8335 (2)	0.5353 (2)	0.0292 (6)	
H1	0.3031	0.7489	0.5769	0.035*	
C2	0.2487 (3)	0.9308 (2)	0.5934 (2)	0.0301 (6)	
H2	0.2322	0.9110	0.6738	0.036*	
C3	0.2320 (3)	1.0577 (2)	0.5330 (2)	0.0262 (5)	
C4	0.2557 (3)	1.0857 (2)	0.4127 (2)	0.0261 (5)	
C5	0.2916 (3)	0.9881 (2)	0.3553 (2)	0.0268 (5)	
H5	0.3037	1.0076	0.2748	0.032*	
C6	0.3103 (3)	0.8606 (2)	0.4157 (2)	0.0258 (5)	
C7	0.3510 (3)	0.7571 (2)	0.3514 (2)	0.0280 (5)	
C8	0.7469 (3)	0.3983 (2)	0.1577 (2)	0.0295 (6)	
H8	0.6571	0.3386	0.1610	0.035*	
C9	0.6777 (3)	0.5199 (2)	0.1690 (2)	0.0297 (6)	
C10	0.8130 (3)	0.6106 (2)	0.1619 (2)	0.0269 (5)	
C11	1.0130 (3)	0.5753 (2)	0.1497 (2)	0.0291 (6)	
H11	1.1030	0.6344	0.1481	0.035*	
C12	1.0809 (3)	0.4523 (2)	0.1397 (2)	0.0286 (5)	
H12	1.2163	0.4292	0.1318	0.034*	
C13	0.9487 (3)	0.3636 (2)	0.1413 (2)	0.0258 (5)	
C14	1.0255 (3)	0.2336 (2)	0.12291 (19)	0.0247 (5)	
C15	0.5818 (4)	0.0203 (2)	0.0254 (2)	0.0299 (6)	
H15A	0.5688	−0.0156	0.1082	0.036*	
H15B	0.7091	−0.0117	−0.0085	0.036*	
C16	0.0738 (4)	0.4632 (3)	0.5389 (2)	0.0377 (6)	
H16A	0.1091	0.5173	0.5853	0.045*	
H16B	0.0145	0.3896	0.5910	0.045*	
N1	0.2550 (3)	0.41936 (18)	0.46971 (18)	0.0344 (5)	
H1B	0.2249	0.3627	0.4335	0.052*	
H1C	0.3452	0.3830	0.5164	0.052*	
H1D	0.3036	0.4862	0.4178	0.052*	
N2	0.5721 (3)	0.16219 (18)	0.00223 (18)	0.0323 (5)	
H2B	0.5803	0.1949	−0.0737	0.048*	
H2C	0.6723	0.1857	0.0298	0.048*	
H2D	0.4579	0.1910	0.0367	0.048*	
O1	0.1944 (3)	1.15782 (15)	0.58542 (14)	0.0342 (4)	
H1A	0.1575	1.1311	0.6545	0.051*	
O2	0.2417 (3)	1.21302 (14)	0.35551 (14)	0.0358 (4)	
H2A	0.2385	1.2187	0.2865	0.054*	
O3	0.3090 (2)	0.77974 (16)	0.24853 (15)	0.0343 (4)	
O4	0.4239 (3)	0.64879 (15)	0.40190 (15)	0.0359 (4)	
O5	0.4788 (3)	0.55228 (19)	0.1832 (2)	0.0518 (6)	
H5A	0.4567	0.6128	0.2145	0.078*	
O6	0.7346 (2)	0.73121 (15)	0.16717 (17)	0.0385 (5)	
H6	0.8245	0.7775	0.1614	0.058*	
O7	1.2060 (2)	0.19869 (15)	0.13369 (14)	0.0319 (4)	
O8	0.9088 (2)	0.16349 (15)	0.09982 (15)	0.0344 (4)	
O1W	−0.0308 (3)	0.90641 (16)	0.18631 (16)	0.0340 (4)	
H1W	0.084 (4)	0.885 (3)	0.208 (2)	0.051*	
H2W	−0.040 (4)	0.990 (2)	0.159 (2)	0.051*	

### Atomic displacement parameters (Å^2^)

**Table:**

	*U*^11^	*U*^22^	*U*^33^	*U*^12^	*U*^13^	*U*^23^
C1	0.0341 (14)	0.0220 (12)	0.0300 (14)	−0.0018 (10)	−0.0076 (11)	−0.0010 (10)
C2	0.0336 (14)	0.0305 (14)	0.0256 (13)	−0.0035 (10)	−0.0042 (11)	−0.0043 (11)
C3	0.0246 (12)	0.0263 (13)	0.0301 (14)	−0.0003 (10)	−0.0049 (10)	−0.0109 (11)
C4	0.0278 (13)	0.0202 (12)	0.0304 (14)	−0.0019 (9)	−0.0088 (10)	−0.0031 (10)
C5	0.0301 (13)	0.0284 (13)	0.0229 (13)	−0.0034 (10)	−0.0069 (10)	−0.0055 (10)
C6	0.0233 (12)	0.0222 (12)	0.0319 (14)	−0.0018 (9)	−0.0053 (10)	−0.0050 (10)
C7	0.0216 (12)	0.0260 (13)	0.0369 (15)	−0.0029 (10)	−0.0032 (11)	−0.0079 (11)
C8	0.0287 (13)	0.0263 (13)	0.0367 (15)	−0.0064 (10)	−0.0054 (11)	−0.0107 (11)
C9	0.0228 (12)	0.0336 (14)	0.0361 (15)	−0.0005 (10)	−0.0051 (11)	−0.0146 (11)
C10	0.0319 (13)	0.0220 (12)	0.0286 (13)	−0.0009 (10)	−0.0073 (10)	−0.0080 (10)
C11	0.0310 (13)	0.0260 (13)	0.0319 (14)	−0.0080 (10)	−0.0073 (11)	−0.0055 (11)
C12	0.0259 (13)	0.0261 (13)	0.0334 (14)	0.0002 (10)	−0.0084 (11)	−0.0041 (11)
C13	0.0271 (12)	0.0258 (12)	0.0250 (13)	−0.0001 (10)	−0.0062 (10)	−0.0057 (10)
C14	0.0304 (13)	0.0235 (12)	0.0191 (12)	−0.0016 (10)	−0.0052 (10)	−0.0016 (10)
C15	0.0289 (13)	0.0294 (13)	0.0317 (14)	0.0011 (10)	−0.0066 (10)	−0.0071 (11)
C16	0.0424 (15)	0.0335 (14)	0.0356 (15)	0.0022 (12)	−0.0085 (12)	−0.0051 (12)
N1	0.0377 (12)	0.0235 (11)	0.0413 (13)	−0.0021 (9)	−0.0100 (10)	−0.0031 (9)
N2	0.0302 (11)	0.0325 (12)	0.0365 (12)	−0.0019 (9)	−0.0078 (9)	−0.0105 (10)
O1	0.0466 (11)	0.0267 (9)	0.0298 (10)	−0.0037 (8)	−0.0008 (8)	−0.0098 (7)
O2	0.0575 (12)	0.0204 (9)	0.0296 (10)	−0.0020 (8)	−0.0139 (9)	−0.0018 (7)
O3	0.0369 (10)	0.0352 (10)	0.0339 (10)	0.0056 (8)	−0.0091 (8)	−0.0151 (8)
O4	0.0416 (10)	0.0203 (9)	0.0457 (11)	0.0010 (7)	−0.0107 (9)	−0.0057 (8)
O5	0.0277 (10)	0.0471 (13)	0.0925 (17)	−0.0009 (8)	−0.0053 (10)	−0.0408 (11)
O6	0.0314 (10)	0.0264 (9)	0.0618 (13)	−0.0010 (7)	−0.0062 (9)	−0.0183 (9)
O7	0.0299 (9)	0.0305 (9)	0.0351 (10)	0.0051 (7)	−0.0080 (8)	−0.0082 (8)
O8	0.0386 (10)	0.0249 (9)	0.0445 (11)	−0.0009 (7)	−0.0168 (8)	−0.0112 (8)
O1W	0.0348 (10)	0.0251 (9)	0.0438 (11)	−0.0016 (8)	−0.0115 (8)	−0.0074 (8)

### Geometric parameters (Å, °)

**Table:**

C1—C2	1.379 (3)	C12—H12	0.9300
C1—C6	1.383 (3)	C13—C14	1.500 (3)
C1—H1	0.9300	C14—O8	1.262 (3)
C2—C3	1.386 (3)	C14—O7	1.268 (3)
C2—H2	0.9300	C15—N2	1.485 (3)
C3—O1	1.362 (3)	C15—C15^i^	1.506 (5)
C3—C4	1.391 (3)	C15—H15A	0.9700
C4—C5	1.376 (3)	C15—H15B	0.9700
C4—O2	1.379 (3)	C16—N1	1.486 (3)
C5—C6	1.393 (3)	C16—C16^ii^	1.507 (5)
C5—H5	0.9300	C16—H16A	0.9700
C6—C7	1.487 (3)	C16—H16B	0.9700
C7—O4	1.265 (3)	N1—H1B	0.8900
C7—O3	1.270 (3)	N1—H1C	0.8900
C8—C9	1.377 (3)	N1—H1D	0.8900
C8—C13	1.389 (3)	N2—H2B	0.8900
C8—H8	0.9300	N2—H2C	0.8900
C9—O5	1.364 (3)	N2—H2D	0.8900
C9—C10	1.401 (3)	O1—H1A	0.8200
C10—O6	1.365 (3)	O2—H2A	0.8200
C10—C11	1.376 (3)	O5—H5A	0.8200
C11—C12	1.385 (3)	O6—H6	0.8200
C11—H11	0.9300	O1W—H1W	0.85 (2)
C12—C13	1.384 (3)	O1W—H2W	0.88 (2)
			
C2—C1—C6	120.6 (2)	C12—C13—C8	119.0 (2)
C2—C1—H1	119.7	C12—C13—C14	119.6 (2)
C6—C1—H1	119.7	C8—C13—C14	121.4 (2)
C1—C2—C3	120.7 (2)	O8—C14—O7	123.1 (2)
C1—C2—H2	119.7	O8—C14—C13	119.3 (2)
C3—C2—H2	119.7	O7—C14—C13	117.6 (2)
O1—C3—C2	123.4 (2)	N2—C15—C15^i^	109.9 (2)
O1—C3—C4	117.6 (2)	N2—C15—H15A	109.7
C2—C3—C4	119.0 (2)	C15^i^—C15—H15A	109.7
C5—C4—O2	122.5 (2)	N2—C15—H15B	109.7
C5—C4—C3	120.1 (2)	C15^i^—C15—H15B	109.7
O2—C4—C3	117.4 (2)	H15A—C15—H15B	108.2
C4—C5—C6	121.0 (2)	N1—C16—C16^ii^	110.7 (3)
C4—C5—H5	119.5	N1—C16—H16A	109.5
C6—C5—H5	119.5	C16^ii^—C16—H16A	109.5
C1—C6—C5	118.6 (2)	N1—C16—H16B	109.5
C1—C6—C7	121.5 (2)	C16^ii^—C16—H16B	109.5
C5—C6—C7	119.9 (2)	H16A—C16—H16B	108.1
O4—C7—O3	122.3 (2)	C16—N1—H1B	109.5
O4—C7—C6	118.7 (2)	C16—N1—H1C	109.5
O3—C7—C6	119.1 (2)	H1B—N1—H1C	109.5
C9—C8—C13	121.0 (2)	C16—N1—H1D	109.5
C9—C8—H8	119.5	H1B—N1—H1D	109.5
C13—C8—H8	119.5	H1C—N1—H1D	109.5
O5—C9—C8	119.9 (2)	C15—N2—H2B	109.5
O5—C9—C10	120.6 (2)	C15—N2—H2C	109.5
C8—C9—C10	119.5 (2)	H2B—N2—H2C	109.5
O6—C10—C11	123.9 (2)	C15—N2—H2D	109.5
O6—C10—C9	116.5 (2)	H2B—N2—H2D	109.5
C11—C10—C9	119.6 (2)	H2C—N2—H2D	109.5
C10—C11—C12	120.3 (2)	C3—O1—H1A	109.5
C10—C11—H11	119.8	C4—O2—H2A	109.5
C12—C11—H11	119.8	C9—O5—H5A	109.5
C13—C12—C11	120.5 (2)	C10—O6—H6	109.5
C13—C12—H12	119.8	H1W—O1W—H2W	108 (2)
C11—C12—H12	119.8		

Symmetry codes: (i) −*x*+1, −*y*, −*z*; (ii) −*x*, −*y*+1, −*z*+1.

### Hydrogen-bond geometry (Å, °)

**Table:**

*D*—H···*A*	*D*—H	H···*A*	*D*···*A*	*D*—H···*A*
N1—H1B···O2^iii^	0.89	2.04	2.904 (3)	163
N1—H1C···O4^iv^	0.89	1.94	2.803 (3)	163
N1—H1D···O4	0.89	1.98	2.741 (3)	143
N2—H2B···O3^v^	0.89	2.09	2.931 (3)	158
N2—H2B···O6^v^	0.89	2.54	3.079 (3)	120
N2—H2C···O8	0.89	1.90	2.742 (2)	157
N2—H2D···O7^vi^	0.89	1.94	2.799 (3)	163
O1—H1A···O1W^vii^	0.82	1.94	2.753 (3)	169
O2—H2A···O7^viii^	0.82	1.95	2.755 (2)	168
O5—H5A···O3	0.82	2.07	2.834 (2)	156
O5—H5A···O4	0.82	2.35	3.014 (3)	139
O6—H6···O1W^ix^	0.82	1.90	2.686 (2)	160
O1W—H1W···O3	0.85 (2)	1.86 (2)	2.676 (2)	159 (3)
O1W—H2W···O8^viii^	0.88 (2)	1.85 (2)	2.725 (2)	173 (3)

Symmetry codes: (iii) *x*, *y*−1, *z*; (iv) −*x*+1, −*y*+1, −*z*+1; (v) −*x*+1, −*y*+1, −*z*; (vi) *x*−1, *y*, *z*; (vii) −*x*, −*y*+2, −*z*+1; (viii) *x*−1, *y*+1, *z*; (ix) *x*+1, *y*, *z*.
